# Zeolitic Imidazolate Framework‑8 (ZIF-8) modified titanium alloy for controlled release of drugs for osteoporosis

**DOI:** 10.1038/s41598-022-13187-0

**Published:** 2022-06-01

**Authors:** Mariusz Sandomierski, Marcel Jakubowski, Maria Ratajczak, Adam Voelkel

**Affiliations:** 1 Institute of Chemical Technology and Engineering, Poznan University of Technology, ul. Berdychowo 4, 60-965 Poznan, Poland; 2 Institute of Building Engineering, Poznan University of Technology, ul. Piotrowo 5, 60-965 Poznan, Poland

**Keywords:** Drug delivery, Biomaterials, Surface chemistry

## Abstract

The aim of this work was to prepare a biocompatible implant material that enables the release of drug for osteoporosis—risedronate. To achieve this goal, a titanium implant coated with a biocompatible Zeolitic Imidazolate Framework 8 (ZIF-8) layer was prepared that promotes osseointegration at the bone-implant interface. The modifications of the titanium alloy as well as sorption and desorption processes were confirmed using a variety of methods: SEM, EDS XPS, and FT-IR imaging (to determine surface modification, drug distribution, and risedronate sorption), and UV–Vis spectroscopy (to determine drug sorption and release profile). Both the ZIF-8 layer and the drug are evenly distributed on the surface of the titanium alloy. The obtained ZIF-8 layer did not contain impurities and zinc ions were strongly bounded by ZIF-8 layer. The ZIF-8 layer was stable during drug sorption. The drug was released in small doses for 16 h, which may help patients recover immediately after surgery. This is the first case of using ZIF-8 on the surface of the titanium alloy as carrier that releases the drug under the influence of body fluids directly at the site of the disease. It is an ideal material for implants designed for people suffering from osteoporosis.

## Introduction

Osteoporosis is the most common bone disease, affecting more than 200 millions of people worldwide^1,2^. The prevalence of osteoporosis increases with age and, as a result, more and more people suffer from it due to aging of the population^3^. Currently used drugs for osteoporosis can be divided into two main groups. The first includes antiresorptive drugs (inhibiting osteoclasts) while the second bone-forming drugs (stimulating osteoblasts)^4^. The most popular antiresorptive drugs used for osteoporosis are bisphosphonates (BPs)^5,6^. Such a wide application of BPs is related to their high selectivity to the bone^7^. Bisphosphonates are ideally suited for the treatment of bone disease because they have a high affinity for hydroxyapatite crystals^8,9^.

Most often, bisphosphonates are taken orally during treatment (in tablet form). Less popular methods of their administration are injections, infusions, and intranasal or transdermal applications. Each of the BP delivery methods used so far has disadvantages. When the drug is administered orally, only a small part of the drug (1–5%) goes to the circulatory system^10^. Oral administration is also associated with many side effects. Some of the many are: heartburn, nausea, irritation of the esophagus, and gastric ulcer. Flu-like symptoms are the main side effects of intravenous administration. Transdermal drug delivery results in local toxicity caused by the release of too much drug over a short period of time^11^. Overall, these drugs are very effective, but better delivery methods are required. One of the directions of new drug release materials for this drug should be implants because osteoporosis is a disease that is often diagnosed too late, so parts of the bone must be replaced^12^. These should be implants that will release the drug in a controlled manner without 'burst release’ to cause local toxicity.

Titanium alloys are commonly used materials in implantology^13^. One of the most widely used is Ti6Al4V, which has very good biocompatibility and low elastic modulus comparable to human bone^14^. Despite the fact that titanium alloys are ideal materials for implant production, it is not possible to use them alone in controlled release of drugs. To produce a material with such properties, the surface of the implant must be previously modified. One type of promising modification is the formation of a biocompatible Zeolitic Imidazolate Framework‑8 (ZIF-8) on their surface^15^.

Zeolitic imidazolate frameworks (ZIFs) are a subclass of metal organic frameworks (MOFs)^16^. ZIFs consist of tetrahedral metal ions (specifically Zn and Co) bridged by imidazolate ligands, and have the advantages of both MOFs and zeolites, such as controllable synthesis and good chemical and thermal stability^17–20^. These properties indicate a potential application for ZIFs in catalysis, adsorption, and separation^21–23^. Moreover, its potential for use in biomedical applications is also attracting increasing attention^24–26^. For example, Liu et al. prepared catechol–chitosan-ZIF-8 hydrogels that promote implantation stability, angiogenesis, and osteogenesis for bone regeneration applications^27^. ZIF nanoparticles were also used as anti-inflammatory and antibacterial platforms to treat periodontitis^28^. In another publication, Sun et al. have reported a pH-sensitive drug delivery system based on ZIFs^29^. ZIFs can also be used in the modification of titanium alloys. Porous titanium modified with nanoscale ZIF-8 coating has been shown to enhance osteogenic and antibacterial activity, increase extracellular matrix mineralization and promotes alkaline phosphatase activity^30^. Subsequent research shows that the ZIF-8 layer enhanced cell bioactivity and also promoted osseointegration at the bone–implant interface^15^. All this information indicates a very high potential for titanium modification with ZIF-8 and the use of this modification in endoprostheses.

**Figure 1:**
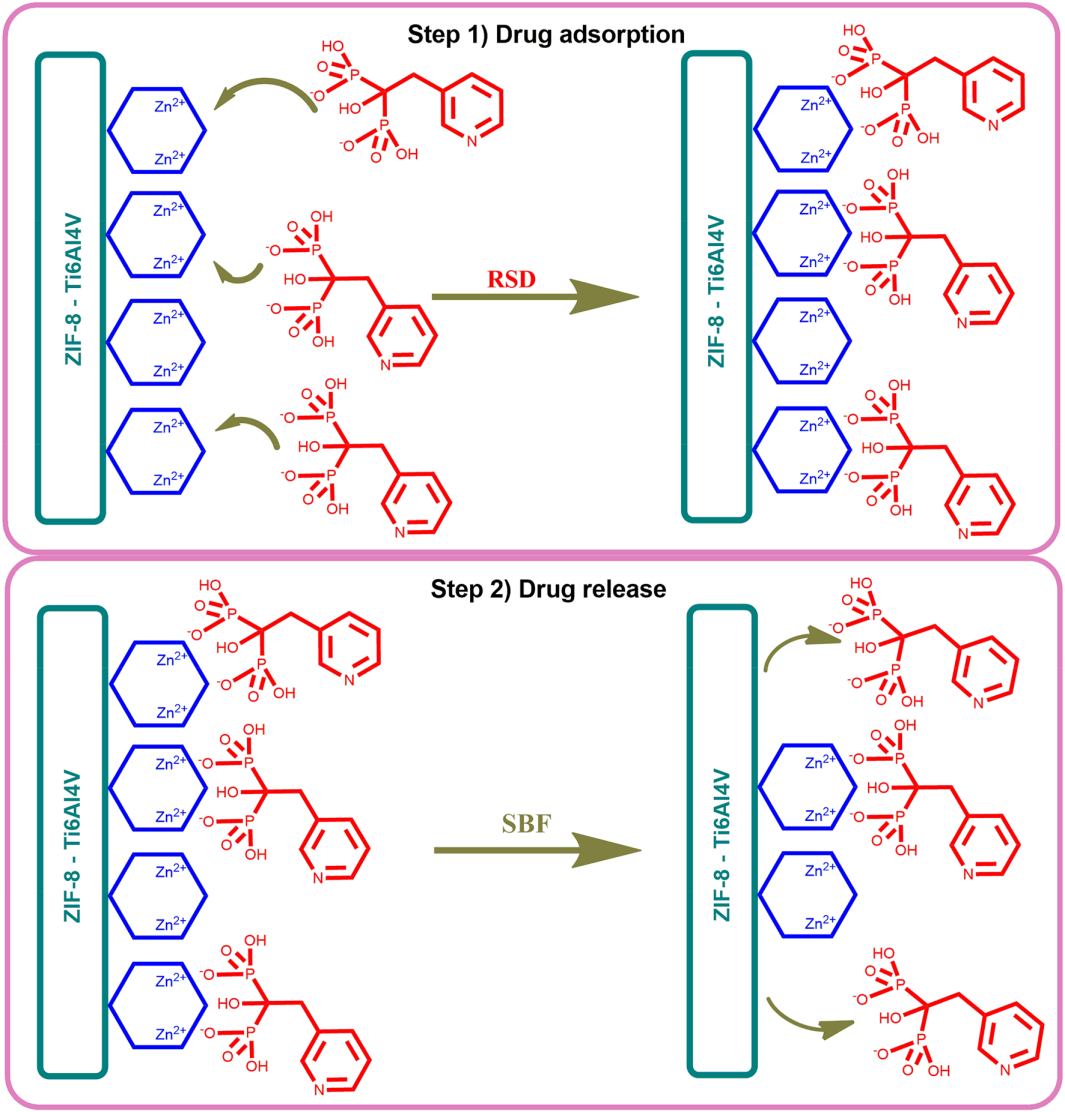
The scheme of the research carried out in this work.

The materials considered during the selection of a carrier in the controlled release of bisphosphonates should have divalent cations in their structure, as we have proven in previous work^31^. This is caused by strong interactions between divalent cations and the phosphonium groups of bisphosphonates^32^. Due to this, a thin layer of ZIF-8 on the surface of the titanium alloys appears to be ideal for the targeted release of bisphosphonates in a place where a bone fragment was needed to be removed. The research scheme of this work is shown in Fig. 1.

In this work, the biocompatible Zeolitic Imidazolate Framework‑8 (ZIF-8) was obtained on the Ti6Al4V alloy surface and used as a risedronate carrier. Two types of alloy modification were used, one previously described and one proposed for the first time in this work. This is the first case of using the Zeolitic Imidazolate Framework 8 (ZIF-8) on the surface of the titanium alloy as carrier that releases the drug under the influence of body fluids directly at the site of the disease. As a result of the deposition of the drug on a biocompatible implant, BPs will be gradually released in the area of greatest demand. Zeolitic Imidazolate Framework‑8 (ZIF-8) on the surface of both Ti6Al4V alloys was characterized to confirm the effectiveness of drug sorption. The sorption capacity and the release rate of risedronate were also examined for the tested materials.

## Materials

### Reagents

Titanium alloy discs—Ti6Al4V (Φ 8 mm, 4 mm thick). Zinc nitrate hexahydrate, 2-methylimidazole (MeIm), sodium hydroxide, tris(hydroxymethyl)aminomethane (TRIS) (99.8%), sodium bicarbonate (99%), potassium chloride (99%), potassium phosphate dibasic trihydrate (99%), sodium sulfate (99%) and sodium risedronate (RSD) were obtained from Sigma-Aldrich. Hydrochloric acid (36–38%) and sodium chloride (99%) were obtained from Avantor. All reagents were used as received without further purification.

### Preparation of ZIF-8 on the surface of the alloy modified with alkali heat treatment (Ti-AHT-ZIF-8)

The methodology of this modification was carried out in accordance with that carried out by Zhang et al.^15^. The titanium discs were sanded, washed with water, ethanol, and acetone. Alkali heat treatment (AHT) of Ti6Al4V was performed according to the procedure described by Chosa et al.^33^. The polished titanium discs were soaked in a 5 M sodium hydroxide aqueous solution at 60 °C for 24 h, followed by washing in distilled water and drying in an oven at 100 °C for 24 h. Subsequently, the discs were heated to 600 °C at a rate of 5 °C/min in an electrical furnace and kept at 600 °C for 1 h. Coating with a ZIF-8 layer on the AHT titanium surface was carried out using a simple and environmentally friendly hydrothermal method. In the first stage of creating the ZIF-8 layer zinc nitrate hexahydrate (0.11 g) and MeIm (2.27 g) were dissolved in 40 ml of deionized water, and stirred for 20 min. The resulting solution was diluted eight times. The solution was then transferred to an autoclave in which the AHT titanium discs were placed and heated at 37 °C for 6 h. Finally, ZIF-8 modified titanium discs were obtained by rinsing with deionized water and drying at 37 °C for 24 h.

The resulting material was named Ti-AHT-ZIF-8.

### Preparation of ZiF-8 on the surface of the alloy modified with zinc titanate (Ti-ZnTit-ZIF-8)

The formation of the ZIF-8 layer on the surface of the titanium alloy presented in this section has not been previously described in the literature. The titanium discs were sanded, washed with water, ethanol, and acetone. Ti6Al4V plates were placed in 5 M sodium hydroxide aqueous solution at 60 °C for a 24 h^34^. The alloy was then washed with distilled water and dried in an oven for 24 h at 100 °C (Ti-NaTit). After drying, the material was placed in a 0.5 M aqueous solution of zinc nitrate for 24 h at room temperature. This process was repeated 3 times. After that, the material was washed with distilled water 3 times and dried in an oven for 24 h at 100 °C (Ti-ZnTit). In the next step, the material was immersed in the MeIm solution for one hour and rinsed again with water. The next steps are analogous to the procedure for Ti6Al4V-AHT-ZIF-8. In the first stage of creating ZIF-8 zinc nitrate hexahydrate (0.11 g) and MeIm (2.27 g) were dissolved in 40 ml of deionized water, and stirred for 20 min. The resulting solution was diluted eight times. The solution was then transferred to an autoclave in which the zinc titanate modified titanium discs were placed, and heated at 37 °C for 6 h. Finally, ZIF-8 modified titanium discs were obtained by rinsing with deionized water and drying at 37 °C for 24 h.

The resulting material was named Ti-ZnTit-ZIF-8.

### Drug sorption

The drug sorption study was initiated by placing modified alloys in Eppendorf tubes filled with 1.5 ml risedronate solution (0.15 mg of risedronate dissolved in 1.5 ml of 0.1 M Tris-HCI). Each sample was placed on an orbital shaker (speed 200 rpm) for one week. The risedronate concentration in solution was tested after 7 days using UV–Vis spectroscopy.

After sorption, Ti-AHT-ZIF-8 was named Ti-AHT-ZIF-8-RSD, while Ti-ZnTit-ZIF-8 was named Ti-ZnTit-ZIF-8-RSD.

### Drug release

The modified plates after risedronate sorption were flooded with 1 ml of simulated body fluid (SBF). The composition of SBF is presented in Table 1. The amount of drug released was measured after each 1 h for up to 16 h using UV–Vis spectroscopy. Each time the SBF was replaced with a new portion. Three repetitions were made for both materials.

**Table 1:** Composition of the simulated body fluid used in this work (1000 ml of the SBF).

Order	Reagent	Amount
1	NaCl	8.035 g
2	NaHCO_3_	0.355 g
3	KCl	0.225 g
4	K_2_HPO_4_·3H_2_O	2.31 g
5	Na_2_SO_4_	0.072 g
6	TRIS	0.6112 g
7	HCl	0–5 ml

## Methods

### Scanning electron microscopy (SEM)/energy dispersive spectroscopy (EDS)

SEM images were recorded with the use of scanning electron microscope VEGA 3 (TESCAN, Czech Republik). The SEM toll was equipped with an EDS analyzer (Bruker, UK). EDS was used to conduct the elemental analysis of the samples. The final concentration of each element is an average value of measurements at 10 point.

### X-ray photoelectron spectroscopy (XPS)

XPS spectra were obtained on a SPECS spectrometer equipped with a monochromatic Al-Kα source emitting photons of energy of 1486.71 eV (XR-50 source with monochromator µ-FOCUS 600) and a hemispherical analyzer (PHOIBOS 150 MCD NAP). The XPS measurement was carried out under an ultrahigh vacuum (UHV) with pressure < 1 × 10^−9^ mbar. The sample was deposited on a sample holder using double-sided adhesive carbon tape. Casa XPS software (version 2.3.24, http://www.casaxps.com/) was used to analyze the recorded spectra.

### Drug distribution evaluation using FT-IR imaging

Drug distribution analysis was performed using a LUMOS II FT-IR microscope (Bruker). The imaging was performed in an area of 1000 × 1000 µm. The distribution of ZIF-8 was determined on the basis of the peaks area which occurs between 1486.3 and 1381.3 cm^−1^. The distribution of drug was determined on the basis of the P–O peak area which is characteristic of risedronate and occurs between 1177.7 and 982.1 cm^−1^. 60 scans were collected for each spectrum. The results were obtained in the reflectance mode. The results were processed using the OPUS 8 software (Bruker).

### UV–Vis spectroscopy

UV–Vis spectrophotometer UV-2600 (Shimadzu, Japan) was applied to determine the concentration of risedronate during the sorption and release process. Measurements were made in the range of 240–305 nm (λ_max_ = 262 nm).

## Results

**Figure 2:**
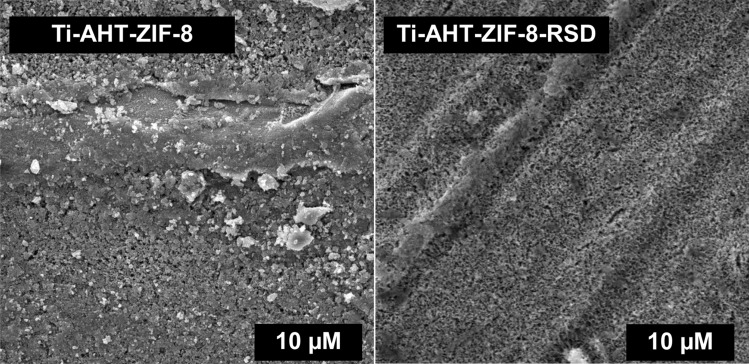
SEM images of Ti-AHT-ZIF-8 and Ti-AHT-ZIF-8-RSD.

The first technique to confirm the effectiveness of surface modification with ZIF-8 was SEM. As can be seen in Fig. 2, ZIF-8 was produced on the titanium surface after the AHT process. Many particles can be seen on the surface, and these results are consistent with those obtained by Zhang et al.^15^. The surface of Ti-AHT-ZIF-8 was compared with the surface after drug adsorption (Ti-AHT-ZIF-8-RSD). There are no more ZIF-8 crystals on the Ti-AHT-ZIF-8-RSD surface, which proves that they were washed away during the sorption process. This is negative information because it indicates that the drug could not be retained on the surface for controlled release.

**Figure 3:**
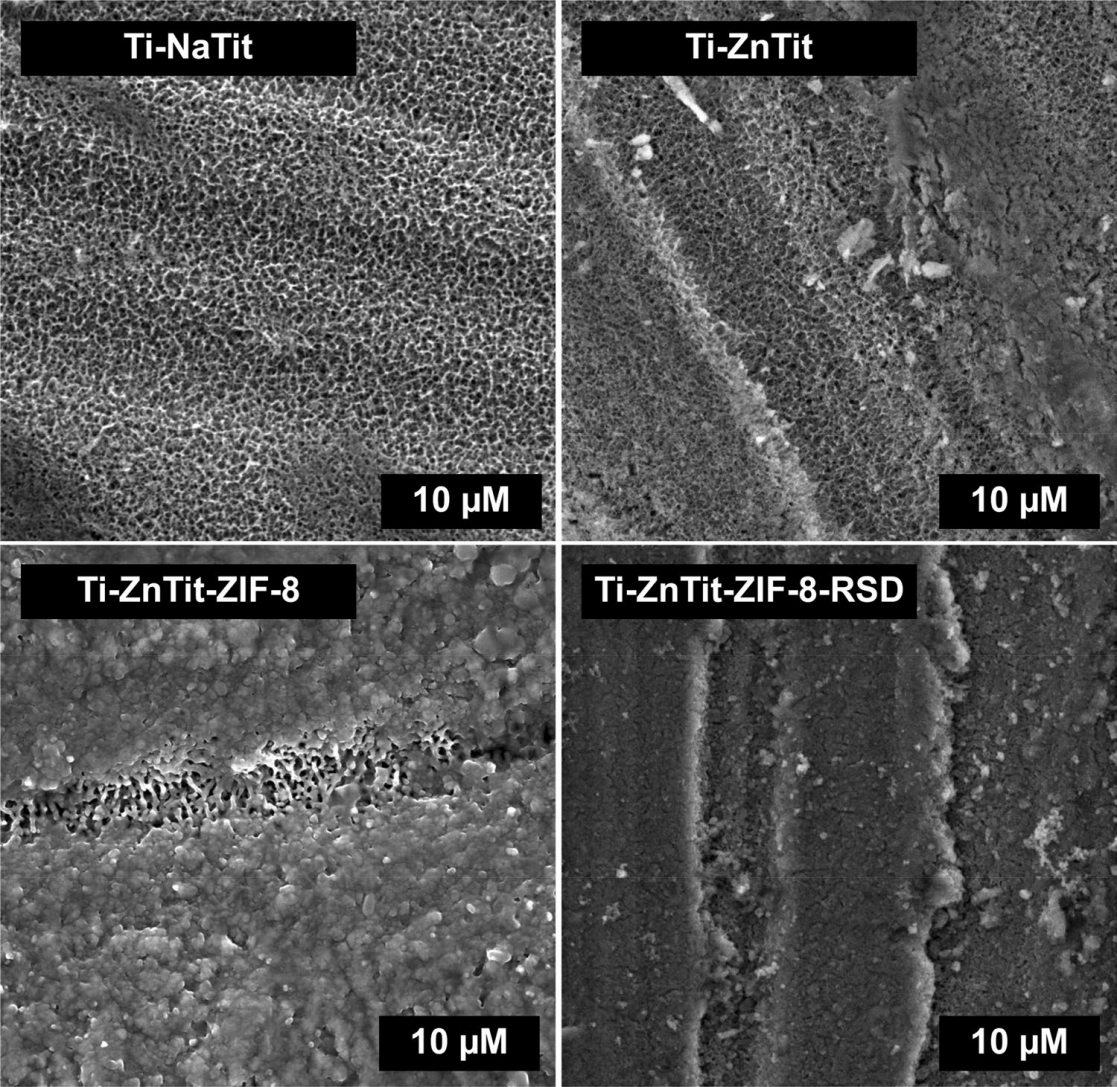
SEM images of Ti-NaTit, Ti-Zn-Tit, Ti-ZnTit-ZIF-8, and Ti-ZnTiT-ZIF-8-RSD.

Using the SEM analysis, the samples produced by the method proposed by our team were also characterized (Fig. 3). The surface of the alloy after alkaline treatment (Ti-NaTit) is very porous, which is consistent with reports from the literature^35,36^. No significant changes were noticed after ion exchange (Ti-ZnTit). Visible changes occur after the formation of the ZIF-8 layer (Ti-ZnTit-ZIF-8). The surface of the alloy is no longer porous, and a very irregular coating is visible on its entire surface. The layer obtained differs significantly from that produced by the first methodology (Ti-AHT-ZIF-8). The structure of this layer is similar to the ZIF-8 layer obtained on Al plates by the ligand-assisted solvothermal conversion of ZnAl-CO3 layered double hydroxide by Zhang et al.^37^. The occurrence of a "crack" in the sample is related to the fact that there was a larger scratch in this place, formed during grinding. This scratch was not completely covered by the ZIF-8 layer. As can be seen on the sample, after sorption of the drug, such a crack does not occur. Importantly, the layer produced by the method proposed by our team also occurs after drug adsorption (Ti-ZnTit-ZIF-8-RSD). The differences between Ti-ZnTit-ZIF-8 and Ti-ZnTit-ZIF-8-RSD are likely due to the drug on the surface Ti-ZnTit-ZIF-8-RSD.

**Figure 4:**
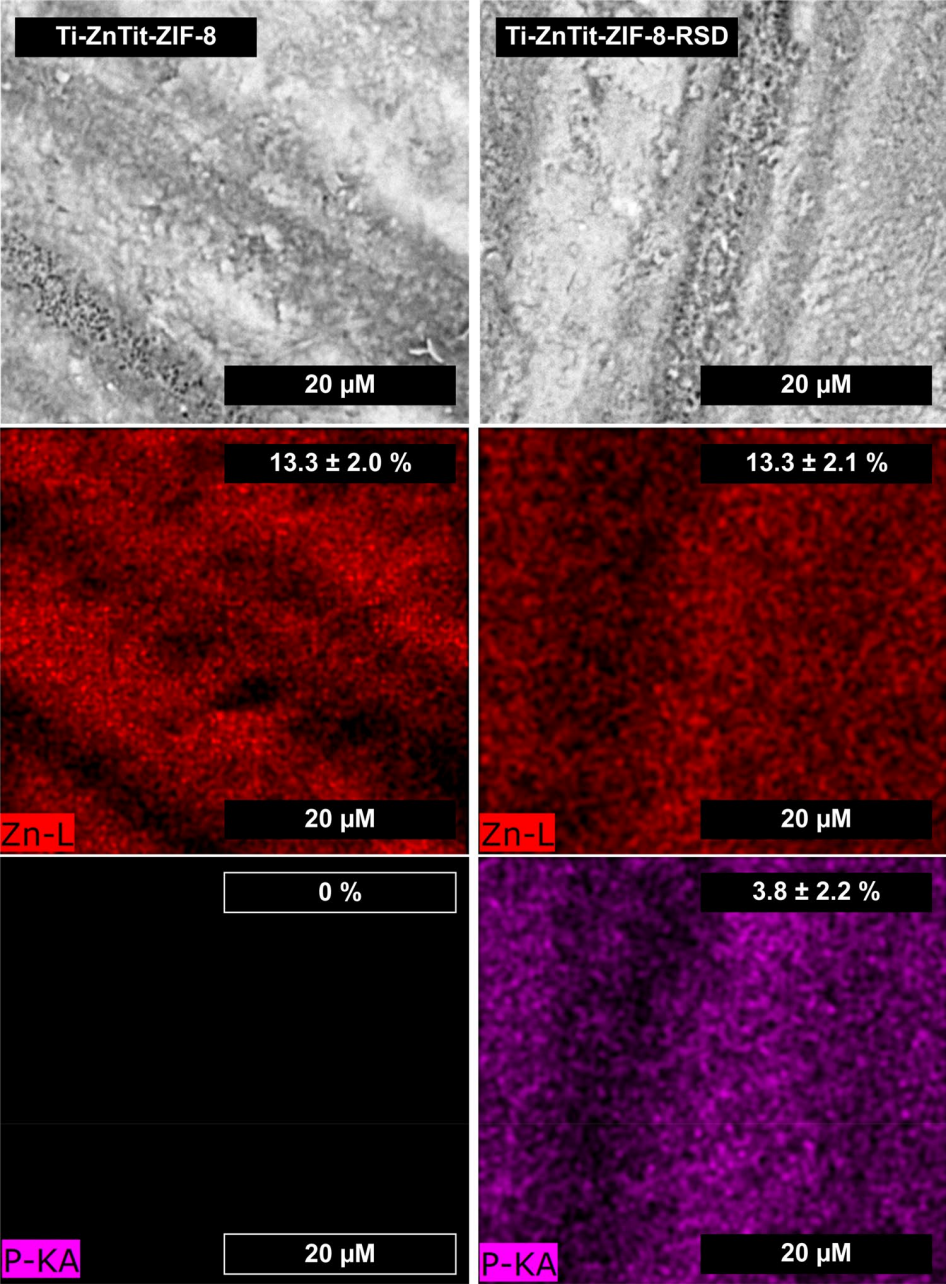
SEM images of alloys (first row). Elemental mapping of the same regions indicating the spatial distribution of zinc (second row) and phosphorus (third row). The values represent the content of elements on the alloy surface (by weight).

The distribution of the layer on the surface of the alloy was also performed using EDS mapping (Fig. 4). As can be seen from the zinc distribution, this layer is visible on the entire surface of the alloy for both Ti-ZnTit-ZIF-8 and Ti-ZnTit-ZIF-8-RSD. The zinc content of both materials is similar. Drug adsorption is proved by the attendance of phosphorous, which is present in the structure of risedronate. One may find it only on the surface of the Ti-Zn-Tit-ZIF-8-RSD sample and is evenly distributed over it.

**Figure 5:**
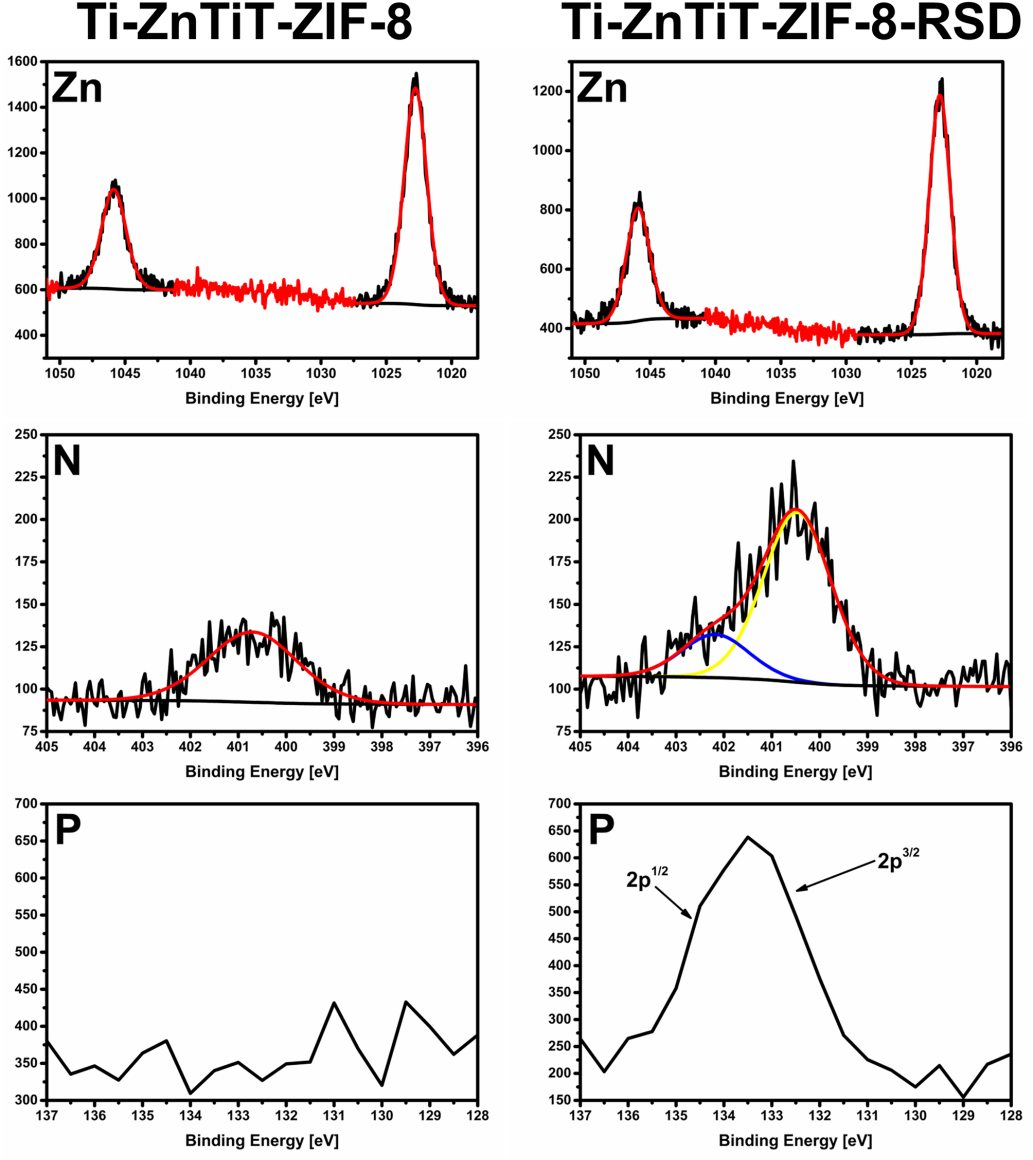
High-resolution XPS spectra of zinc, nitrogen, and phosphorous for the Ti ZnTit-ZIF-8 and Ti-ZnTit-ZIF-8-RSD samples.

**Figure 6:**
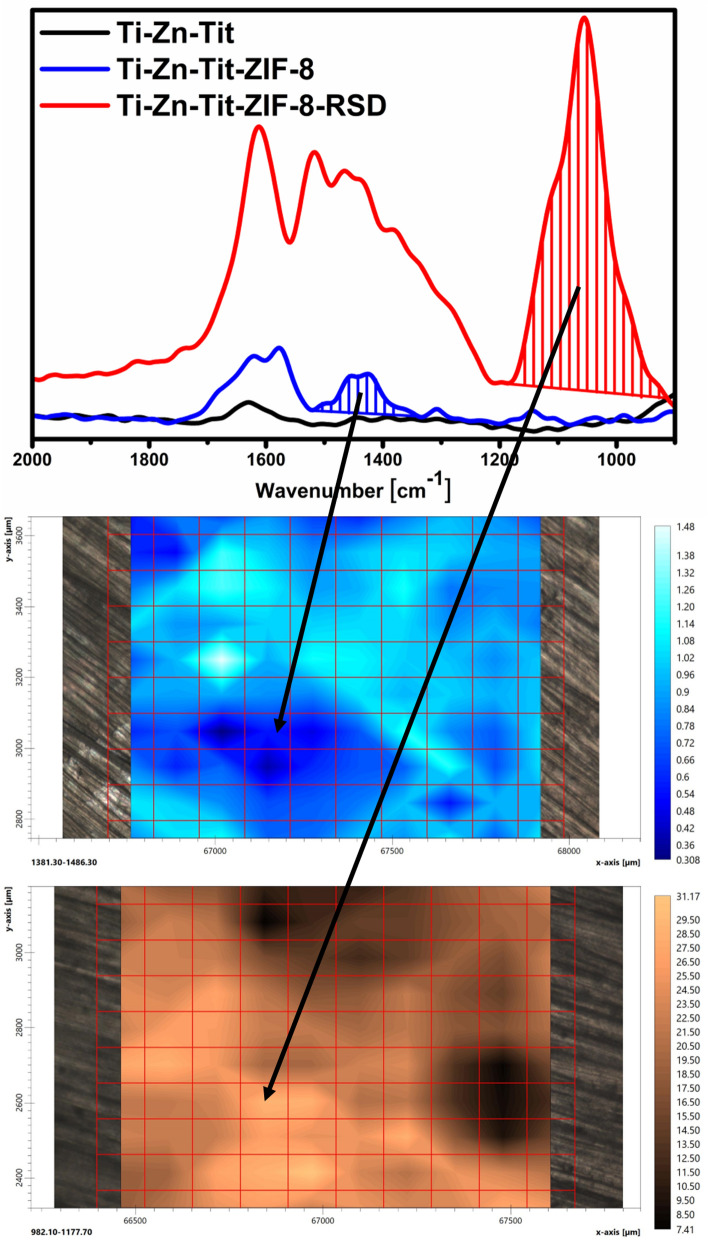
ZIF-8 layer and risedronate distribution using FT-IR imaging, and band for which the images were prepared (top).

The presence of zinc in both samples was also confirmed by XPS analysis (Fig. 5). The Zn binding energy range shows peaks at 1023.2 eV that correspond to Zn 2p^3/2^, and peaks at a higher binding energy of 1045.9 eV associated with Zn 2p^1/2^^38^. The ~ 23.1 eV difference between Zn 2p^3/2^ and Zn 2p^1/2^ indicates the oxidation state + 2 for Zn ions in both samples and this was not changed after drug sorption^39,40^. The presence of nitrogen on the surface of the Ti-ZnTit-ZIF-8 sample indicates the effectiveness of the ZIF-8 layer formation. A narrow symmetric peak in the N 1 s XPS spectra of ZIF-8 indicates that there is only one form of nitrogen in the framework. The presence of only one peak indicates that there are no defects in the obtained layer and that all rings are coordinated by zinc^41^. Furthermore, the peak for nitrogen shifts to a higher binding energy than that for nitrogen in the free molecule, also indicating that it is bound to the metal^42,43^. Two nitrogen peaks are visible after drug sorption. One can be assigned to the ZIF-8 layer and the other to the drug. These peaks indicate the presence of C = N/C–N bonds^44,45^. The peak for P observed in the XPS spectra of Ti-ZnTit-ZIF-8-RSD clearly indicates the effectiveness of drug sorption (Fig. 6). The deconvolution of high-resolution P 2p spectra for Ti-ZnTit-ZIF-8-RSD shows a characteristic asymmetric P 2p^1/2^ and P 2p^3/2^, confirming the presence of risedronate on the surface^46,47^. For Ti-ZnTit-ZIF-8, these peaks do not exist.

The distribution of the ZIF-8 layer, and drug was determined using FT-IR imaging. As can be seen in Fig. 6, both are present over the entire surface of the material. The bands for which the ZIF-8 and drug distribution were determined are not present in Ti-Zn-Tit material. The imaging of the ZIF-8 layer was performed for bands in the range 1486.3 and 1381.3 cm^−1^ that can be assigned to the imidazole ring stretching^48,49^. The imaging of the drug distribution was performed for P–O band in the range 1177.7 and 982.1 cm^−1^, which clearly confirms the presence of the drug^50^. These bands also occur after drug sorption, which indirectly means that the ZIF-8 layer has not been removed. The even distribution of the ZIF-8 and risedronate that has been achieved on the surface of this material is very important because the drug should be delivered from the surface of the endoprosthesis to the same extent at each point of contact with the tissues.

**Figure 7:**
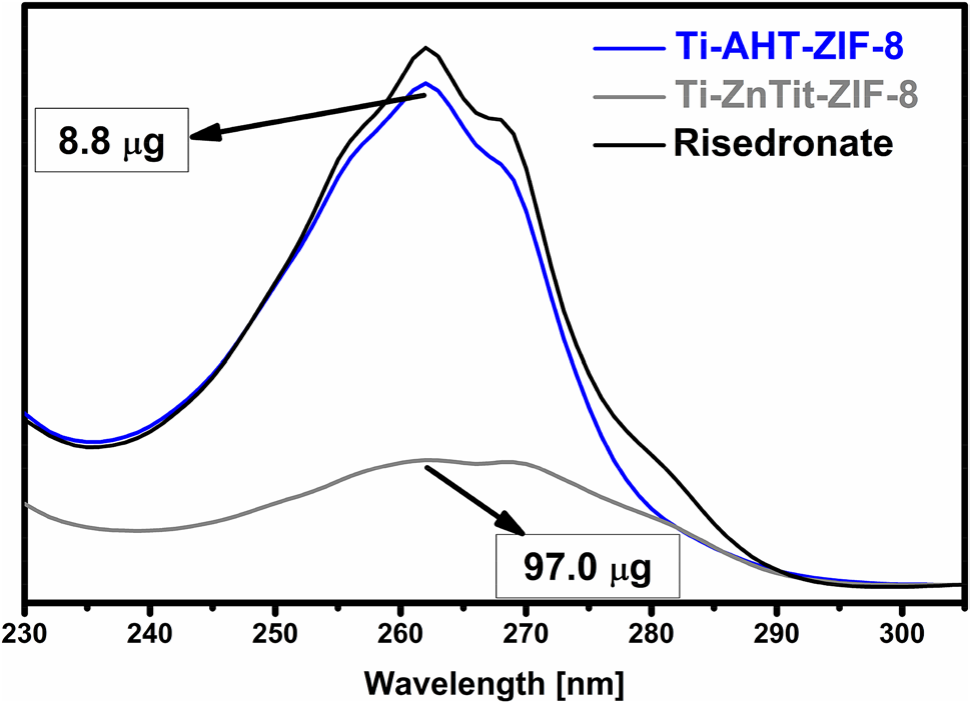
Risedronate sorption after 7 days on the surface of modified Ti6Al4V alloys (“Risedronate” means the starting concentration).

UV/Vis spectroscopy was used to determine the exact amount of drug retained by the modified alloys. The results are presented in Fig. 7. The titanium alloy modified with the method proposed in this study retained more than 10 times more drug.

**Figure 8:**
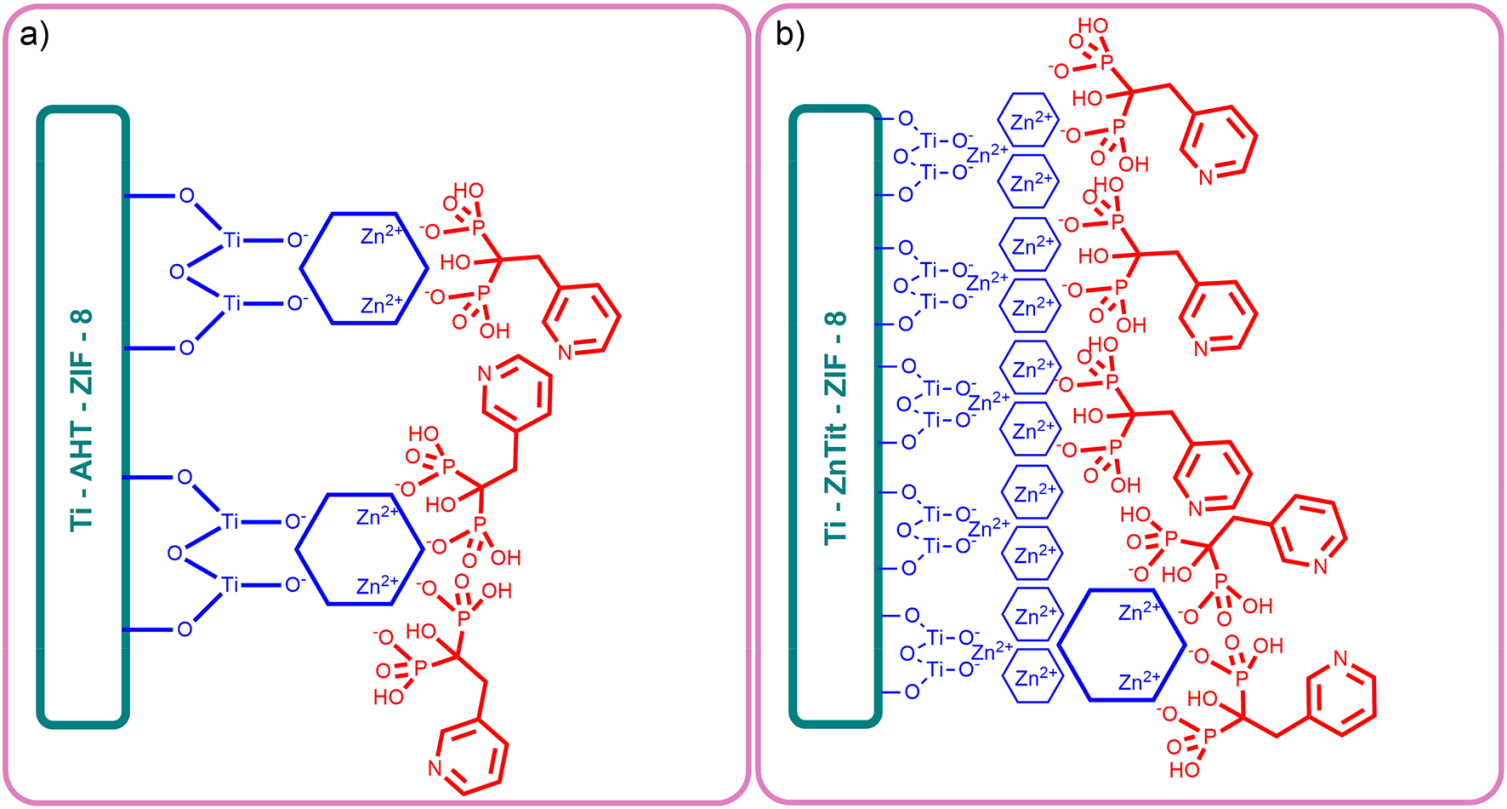
Potential scheme of drug adsorption on the surface of alloys modified with two methods.

The potential reason for the high effectiveness of the second material is presented in Fig. 8. Probably, there are only single ZIF-8 crystals on the surface of the Ti-AHT-ZIF-8 material that was visible in the SEM analysis. Because of the low availability of zinc ions in such large crystals, the drug has limited ability to attach. On the other hand, an evenly distributed layer of ZIF-8 is formed on the surface of the entire Ti-ZnTit-ZIF-8 material. Because of this, it is possible to attach a lot more drug to its surface. In addition, the ZIF-8 layer is connected to the alloy surface by zinc ions from zinc titanate, which contributes to its stability on the surface.

**Figure 9:**
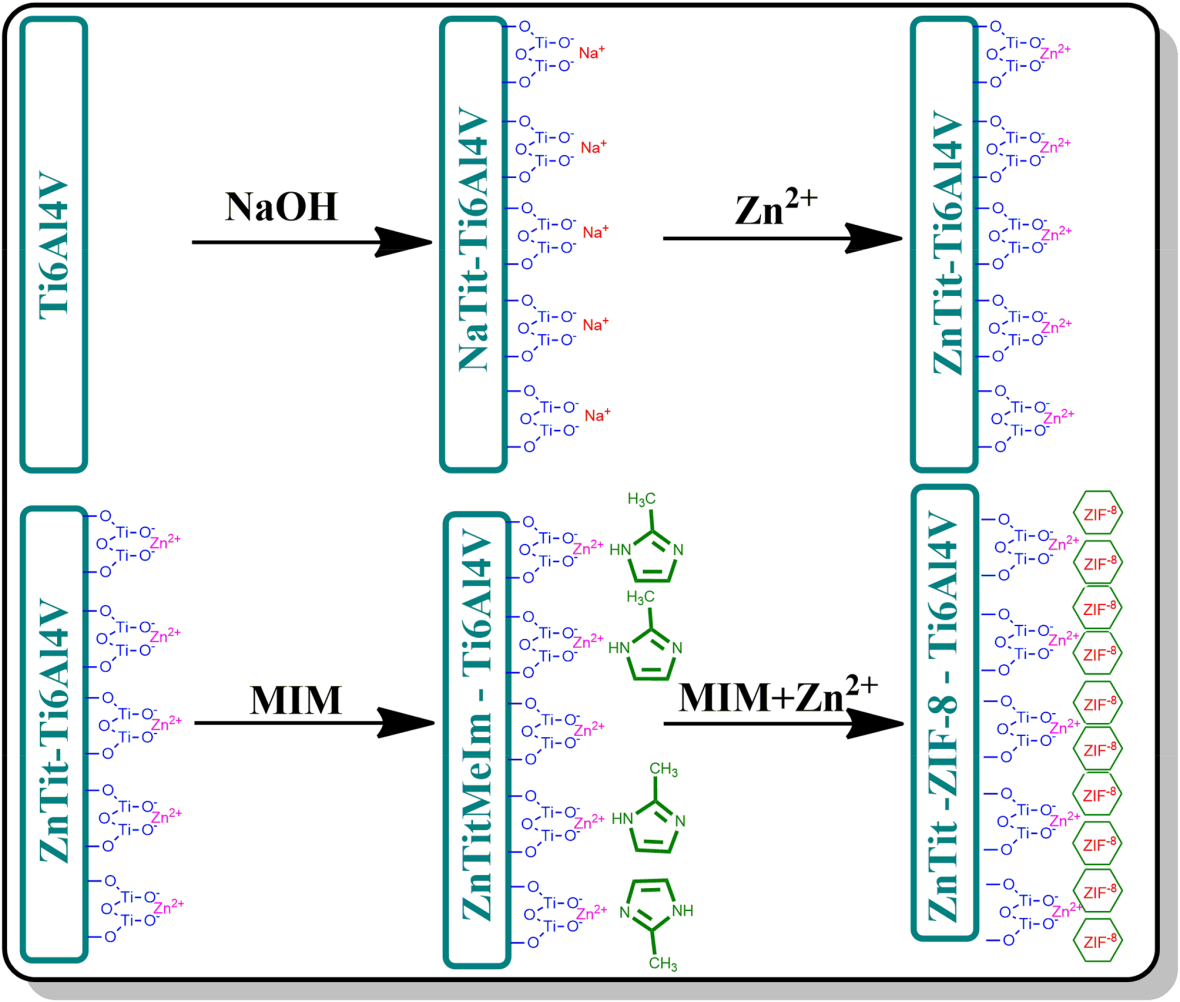
Detailed mechanism of the ZIF-8 layer formation on the surface of a titanium alloy modified with zinc titanate.

To explain in more detail why the modification presented in this article allows for the creation of a ZIF-8 layer that is more stable and distributed over the entire surface, one should look at the process scheme shown in Fig. 9. In the first stage, a typical modification of a titanium alloy was used, in which sodium titanate is produced on its surface under the influence of sodium hydroxide. In the second stage, sodium ions are exchanged for zinc ions in the process of ion exchange. The exchange with zinc ions was carried out because they participate in the crystallization of ZIF-8 and it was assumed that they could contribute to greater adhesion of the produced ZIF-8 layer to titanium. In the next step, the plates were placed in the MeIm solution. This step was to create a thin MeIm layer, which was also to contribute to increasing the adhesion between the alloy and the ZIF-8 layer.

**Figure 10:**
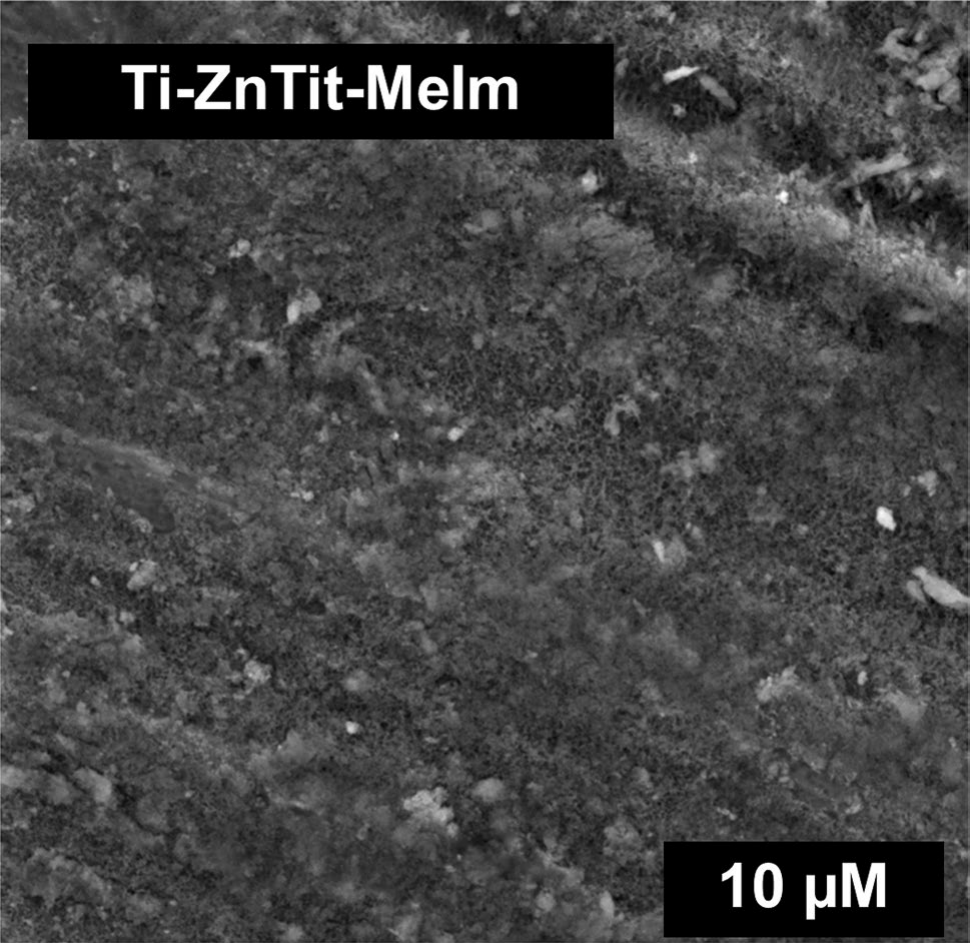
SEM image of Ti-ZnTit-MeIm.

Figure 10 shows that the ZIF-8 structure (which is shown in Fig. 3) is not yet formed on the alloy surface after this step. There are some changes compared to zinc titanate layer, but they are minor. This layer initiates the build-up of ZIF-8 on the surface of the titanium alloy. The final step was to place the plate in a solution of MeIm and zinc ions. All of these steps contributed to the creation of a layer that is more stable and allows the retention of an incomparably greater amount of the drug.

**Figure 11:**
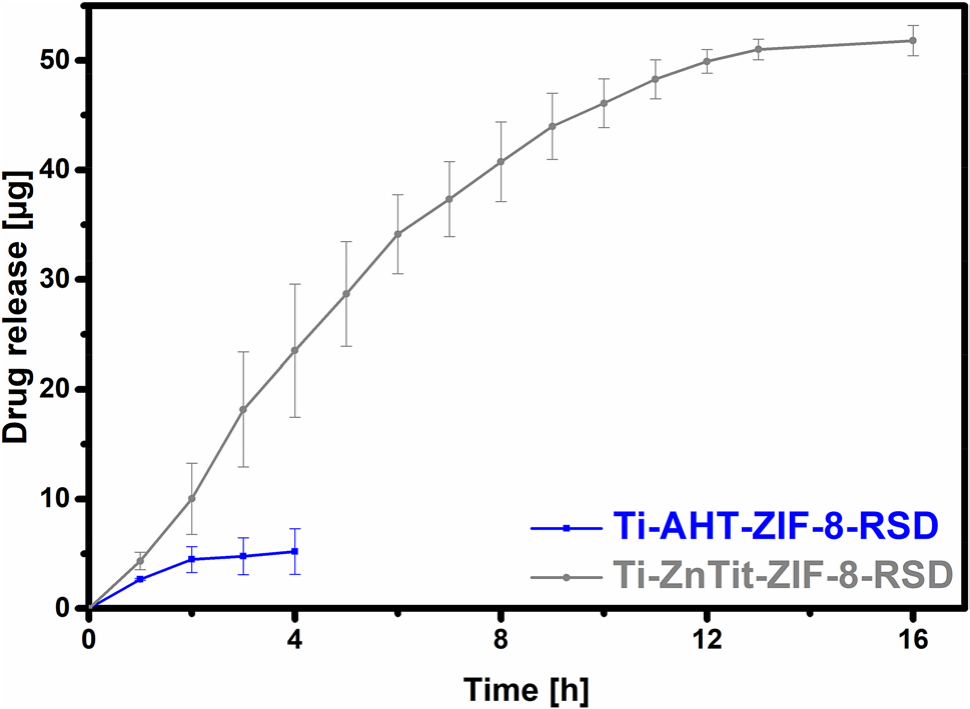
Total release of risedronate from the surface of Ti6Al4V alloys coated with ZIF-8.

Retaining the desired amount of drug on the surface is the first step in the preparation of the drug carrier and is not the most important step. More important is how much medicine is released and at what time. The release results are presented in Fig. 11. Drug release from the Ti-AHT-ZIF-8-RSD surface was very short and a small amount of drug was released (5.2 µg). The situation is different in the case of Ti-ZnTit-ZIF-8-RSD. The release continued for as long as 16 h (after this time, the drug was undetectable) and 52.4 µg was released from 0.5 cm^2^. The surfaces of endoprostheses are several hundred times larger, which will allow the selection of the appropriate dose of the drug for a given application. Importantly, the drug was released in small doses, which proves that the alloy will not cause toxic reactions. On the basis of the results obtained, it can be assumed that the presented material is suitable for supporting the recovery of people suffering from osteoporosis immediately after surgery.

## Conclusions

In the presented work, we created a stable and biocompatible layer of ZIF-8 on the surface of a titanium alloy by modifying the previously described method^15^. We attached a drug for osteoporosis to the created layer. Both the ZIF-8 layer obtained with our method and the drug on its surface were evenly distributed across the entire surface of the alloy, which was confirmed by EDS mapping, and FTIR imaging. The results of the XPS analysis show that the layer obtained by our method has no defects or impurities in the form of unbound imidazole rings. Comparing our material to that described in the literature, it was found that it retains 10 times more of the drug. Additionally, our material releases a much larger dose of the drug within 16 h. Due to its biocompatibility and enhanced osseointegration, the material described in this work is a potential material for use as an element of endoprostheses. Due to the fact that it will also release the drug after the operation, it will increase the chances of a quick recovery of the patient. Furthermore, the proposed implant also has zinc ions that have antibacterial properties.

## Data Availability

The datasets generated during and/or analysed during the current study are available from the corresponding author on reasonable request.

## References

[1] Pavone V, Testa G, Giardina SMC, Vescio A, Restivo DA, Sessa G (2017). Pharmacological therapy of osteoporosis: A systematic current review of literature. Front. Pharmacol..

[2] Ensrud KE, Crandall CJ (2017). Osteoporosis. Ann. Intern. Med..

[3] De Martinis M, Di Benedetto MC, Mengoli LP, Ginaldi L (2006). Senile osteoporosis: Is it an immune-mediated disease?. Inflamm. Res..

[4] De Martinis M, Sirufo MM, Ginaldi L (2020). Osteoporosis: Current and emerging therapies targeted to immunological checkpoints. Curr. Med. Chem..

[5] Kanis JA, Gluer CC (2000). An update on the diagnosis and assessment of osteoporosis with densitometry. Osteoporos. Int..

[6] Russell RGG, Watts NB, Ebetino FH, Rogers MJ (2008). Mechanisms of action of bisphosphonates: Similarities and differences and their potential influence on clinical efficacy. Osteoporos. Int..

[7] Graham R, Russell G (2011). Bisphosphonates: The first 40 years. Bone.

[8] Green JR (2004). Bisphosphonates: Preclinical review. Oncologist.

[9] Woo S-B, Hellstein JW, Kalmar J (2006). Systematic review: Bisphosphonates and osteonecrosis of the jaws. Ann. Intern. Med..

[10] Bartl R, Frisch B, von Tresckow E, Bartl C (2007). Bisphosphonates in Medical Practice: Actions-Side Effects-Indications-Strategies.

[11] Giger EV, Castagner B, Leroux JC (2013). Biomedical applications of bisphosphonates. J. Control. Release.

[12] Fini M, Giavaresi G, Torricelli P, Borsari V, Giardino R, Nicolini A, Carpi A (2004). Osteoporosis and biomaterial osteointegration. Biomed. Pharmacother..

[13] Sandomierski M, Buchwald T, Voelkel A (2020). The possibility of the polyurethane layer attachment to the unmodified and diazonium-modified titanium alloy applied as potential biomaterial. Surf. Coat. Technol..

[14] Liu X, Chu PK, Ding C (2004). Surface modification of titanium, titanium alloys, and related materials for biomedical applications. Mater. Sci. Eng. R.

[15] Zhang X, Chen J, Pei X, Wang J, Wan Q, Jiang S, Huang C, Pei X (2017). Enhanced osseointegration of porous titanium modified with zeolitic imidazolate framework-8. ACS Appl. Mater. Interfaces.

[16] Yaghi OM, O’Keeffe M, Ockwig NW, Chae HK, Eddaoudi M, Kim J (2003). Reticular synthesis and the design of new materials. Nature.

[17] Tian Y, Chen Z, Weng L, Guo H, Gao S, Zhao DY (2004). Two polymorphs of Cobalt(II) imidazolate polymers synthesized solvothermally by using one organic template *N*,*N*-dimethylacetamide. Inorg. Chem..

[18] Banerjee R, Furukawa H, Britt D, Knobler C, O’Keeffe M, Yaghi OM (2009). Control of pore size and functionality in isoreticular zeolitic imidazolate frameworks and their carbon dioxide selective capture properties. J. Am. Chem. Soc..

[19] Mccarthy MC, Varelaguerrero V, Barnett GV, Jeong HK (2010). Synthesis of zeolitic imidazolate framework films and membranes with controlled microstructures. Langmuir.

[20] Fairen-Jimenez D, Moggach SA, Wharmby MT, Wright PA, Parsons S, Duren T (2011). Opening the gate: Framework flexibility in Zif-8 explored by experiments and simulations. J. Am. Chem. Soc..

[21] Chizallet C, Lazare S, Bazer-Bachi D, Bonnier F, Lecocq V, Soyer E, Quoineaud AA, Bats N (2010). Catalysis of transesterification by a nonfunctionalized metal-organic framework: Acido-basicity at the external surface of Zif-8 probed by Ftir and ab initio calculations. J. Am. Chem. Soc..

[22] Wang B, Côté AP, Furukawa H, O’Keeffe M, Yaghi OM (2008). Colossal cages in zeolitic imidazolate frameworks as selective carbon dioxide reservoirs. Nature.

[23] Venna SR, Carreon MA (2010). Highly permeable zeolite imidazolate framework-8 membranes for CO_2_/CH_4_ separation. J. Am. Chem. Soc..

[24] Furukawa H, Cordova KE, O’Keeffe M, Yaghi OM (2013). The chemistry and applications of metal-organic frameworks. Science.

[25] Della Rocca J, Liu D, Lin W (2011). Nanoscale metal-organic frameworks for biomedical imaging and drug delivery. Acc. Chem. Res..

[26] Wang Q, Sun Y, Li S, Zhang P, Yao Q (2020). Synthesis and modification of ZIF-8 and its application in drug delivery and tumor therapy. RSC Adv..

[27] Liu Y, Zhu Z, Pei X, Zhang X, Cheng X, Hu S, Gao X, Wang J, Chen J, Wan Q (2020). ZIF-8-modified multifunctional bone-adhesive hydrogels promoting angiogenesis and osteogenesis for bone regeneration. ACS Appl. Mater. Interfaces.

[28] Li X, Qi M, Li C, Dong B, Wang J, Weir MD, Imazato S, Du L, Lynch CD, Xu L, Zhou Y, Wang L, Xu HHK (2019). Novel nanoparticles of cerium-doped zeolitic imidazolate frameworks with dual benefits of antibacterial and anti-inflammatory functions against periodontitis. J. Mater. Chem. B.

[29] Sun CY, Qin C, Wang XL, Yang GS, Shao KZ, Lan YQ, Su ZM, Huang P, Wang CG, Wang EB (2012). Zeolitic imidazolate framework-8 as efficient Ph-sensitive drug delivery vehicle. Dalton Trans..

[30] Chen J, Zhang X, Huang C, Cai H, Hu S, Wan Q, Pei X, Wang J (2017). Osteogenic activity and antibacterial effect of porous titanium modified with metal-organic framework films. J. Biomed. Mater. Res. Part A.

[31] Sandomierski M, Zielińska M, Voelkel A (2020). Calcium zeolites as intelligent carriers in controlled release of bisphosphonates. Int. J. Pharm..

[32] Gałęzowska J (2018). Interactions between clinically used bisphosphonates and bone mineral: From coordination chemistry to biomedical applications and beyond. ChemMedChem.

[33] Chosa N, Taira M, Saitoh S, Sato N, Araki Y (2004). Characterization of apatite formed on alkaline-heat-treated Ti. J. Dent. Res..

[34] Kokubo T, Yamaguchi S (2010). Novel bioactive titanate layers formed on Ti metal and its alloys by chemical treatments. Materials.

[35] Carradò A, Perrin-Schmitt F, Le QV, Giraudel M, Fischer C, Koenig G, Jacomine L, Behr L, Chalom A, Fiette L, Morlet A, Pourroy G (2017). Nanoporous hydroxyapatite/sodium titanate bilayer on titanium implants for improved osteointegration. Dent. Mater..

[36] Wang H, Lai YK, Zheng RY, Bian Y, Zhang KQ, Lin CJ (2015). Tuning the surface microstructure of titanate coatings on titanium implants for enhancing bioactivity of implants. Int. J. Nanomed..

[37] Zhang M, Ma L, Wang L, Sun Y, Liu Y (2018). Insights into the use of metal–organic framework as high-performance anticorrosion coatings. ACS Appl. Mater. Interfaces.

[38] Muñoz-Gil D, Figueiredo FML (2019). High surface proton conduction in nanostructured ZIF-8. Nanomaterials.

[39] Li PZ, Aranishi K, Xu Q (2012). ZIF-8 immobilized nickel nanoparticles: Highly effective catalysts for hydrogen generation from hydrolysis of ammonia borane. Chem. Commun..

[40] Liu J, He J, Wang L, Li R, Chen P, Rao X, Deng L, Rong L, Lei J (2016). NiO-PTA supported on ZIF-8 as a highly effective catalyst for hydrocracking of Jatropha oil. Sci. Rep..

[41] Tian F, Cerro AM, Mosier AM, Wayment-Steele HK, Shine RS, Park A, Webster ER, Johnson LE, Johal MS, Benz L (2014). Surface and stability characterization of a nanoporous ZIF-8 thin film. J. Phys. Chem. C.

[42] Gadipelli S, Travis W, Zhou W, Guo Z (2014). A thermally derived and optimized structure from ZIF-8 with giant enhancement in CO_2_ uptake. Energy Environ. Sci..

[43] Artyushkova K, Kiefer B, Halevi B, Knop-Gericke A, Schlogl R, Atanassov P (2013). Density functional theory calculations of XPS binding energy shift for nitrogen-containing graphene-like structures. Chem. Commun..

[44] Bronze-Uhle ES, Dias LFG, Trino LD, Matos AA, de Oliveira RC, Lisboa-Filho PN (2019). Physicochemical bisphosphonate immobilization on titanium dioxide thin films surface by UV radiation for bio-application. Surf. Coat. Technol..

[45] Metoki N, Liu L, Beilis E, Eliaz N, Mandler D (2014). Preparation and characterization of alkylphosphonic acid self-assembled monolayers on titanium alloy by chemisorption and electrochemical deposition. Langmuir.

[46] Mc Leod K, Kumar S, Smart RSC, Dutta N, Voelcker NH, Anderson GI, Sekel R (2006). XPS and bioactivity study of the bisphosphonate pamidronate adsorbed onto plasma sprayed hydroxyapatite coatings. Appl. Surf. Sci..

[47] Rudzka K, Trevino AYS, Rodríguez-Valverde MA, Cabrerizo-Vílchez MA (2016). Formation of mixed and patterned self-assembled films of alkylphosphonates on commercially pure titanium surfaces. Appl. Surf. Sci..

[48] Hu Y, Kazemian H, Rohani S, Huang Y, Song Y (2011). In situ high pressure study of ZIF-8 by FTIR spectroscopy. Chem. Comm..

[49] Md Nordin NAH, Racha SM, Matsuura T, Misdan N, Sani NAA, Ismail AF, Mustafa A (2015). Facile modification of ZIF-8 mixed matrix membrane for CO_2_/CH_4_ separation: Synthesis and preparation. RSC Adv..

[50] Sahana H, Khajuria DK, Razdan R, Roy Mahapatra D, Bhat MR, Suresh S, Rao RR, Mariappan L (2013). Improvement in bone properties by using risedronate adsorbed hydroxyapatite novel nanoparticle based formulation in a rat model of osteoporosis. J. Biomed. Nanotechnol..

